# Interaction of the Molecular Chaperone DNAJB6 with Growing Amyloid-beta 42 (Aβ42) Aggregates Leads to Sub-stoichiometric Inhibition of Amyloid Formation*

**DOI:** 10.1074/jbc.M114.595124

**Published:** 2014-09-12

**Authors:** Cecilia Månsson, Paolo Arosio, Rasha Hussein, Harm H. Kampinga, Reem M. Hashem, Wilbert C. Boelens, Christopher M. Dobson, Tuomas P. J. Knowles, Sara Linse, Cecilia Emanuelsson

**Affiliations:** From the ‡ Department of Biochemistry & Structural Biology, Center for Molecular Protein Science, Lund University, PO box 124, 221 00 Lund, Sweden,; § Department of Chemistry, University of Cambridge, Lensfield Road, CB2 1EW, Cambridge, United Kingdom,; ¶ Department of Cell Biology, UMCG and RuG, Groningen, The Netherlands,; ‖ Department of Biochemistry, Faculty of Pharmacy, Beni-Suef University, Salah Salem Street, 62511 Beni-Suef, Egypt, and; ** Department of Biomolecular Chemistry, Radboud University Nijmegen, PO Box 9101, 6500 HB Nijmegen, The Netherlands

**Keywords:** Alzheimer Disease, Amyloid-beta (Aβ), Chaperone DnaJ (DnaJ), Neurodegenerative Disease, Protein Aggregation, Hsp40, Aggregation Kinetics, Amyloid Fibril Formation, Inhibition Mechanism

## Abstract

The human molecular chaperone protein DNAJB6 was recently found to inhibit the formation of amyloid fibrils from polyglutamine peptides associated with neurodegenerative disorders such as Huntington disease. We show in the present study that DNAJB6 also inhibits amyloid formation by an even more aggregation-prone peptide (the amyloid-beta peptide, Aβ42, implicated in Alzheimer disease) in a highly efficient manner. By monitoring fibril formation using Thioflavin T fluorescence and far-UV CD spectroscopy, we have found that the aggregation of Aβ42 is retarded by DNAJB6 in a concentration-dependent manner, extending to very low sub-stoichiometric molar ratios of chaperone to peptide. Quantitative kinetic analysis and immunochemistry studies suggest that the high inhibitory efficiency is due to the interactions of the chaperone with aggregated forms of Aβ42 rather than the monomeric form of the peptide. This interaction prevents the growth of such species to longer fibrils and inhibits the formation of new amyloid fibrils through both primary and secondary nucleation. A low dissociation rate of DNAJB6 from Aβ42 aggregates leads to its incorporation into growing fibrils and hence to its gradual depletion from solution with time. When DNAJB6 is eventually depleted, fibril proliferation takes place, but the inhibitory activity can be prolonged by introducing DNAJB6 at regular intervals during the aggregation reaction. These results reveal the highly efficacious mode of action of this molecular chaperone against protein aggregation, and demonstrate that the role of molecular chaperones can involve interactions with multiple aggregated species leading to the inhibition of both principal nucleation pathways through which aggregates are able to form.

## Teaser

**Background:** The origins of the inhibition of DNAJB6 against amyloid formation are unknown.

**Results:** DNAJB6 inhibits fibril formation of the Aβ42 peptide from Alzheimer disease at low sub-stoichiometric molar ratios through strong binding to aggregated species.

**Conclusion:** Such sequestration prevents the growth and the proliferation of the aggregates.

**Significance:** The efficacious action of the chaperone against amyloid formation involves interactions with multiple growing aggregates.

## Introduction

Amyloid fibrils are highly ordered protein aggregates characterized by a repeating substructure of β-strands oriented perpendicularly to the fiber axis, an arrangement which gives rise to a characteristic cross-β diffraction pattern (1, 2). Since the amyloid state is characterized by a dense network of hydrogen bonding connecting groups within the polypeptide backbone common to all peptides and proteins, the ability to form amyloid fibrils is a generic property of polypeptides (3), although the propensity to form fibrils under specific conditions is modulated by the amino acid sequence (4, 5).

Over 40 human proteins are found in amyloid deposits associated with human diseases, including Huntington and Alzheimer disease (1, 6). Alzheimer disease (AD)^2^ is one of the most common forms of dementia affecting over 40 million people worldwide. Hallmarks of this disease include extracellular amyloid deposits containing the amyloid β peptide (Aβ) and intracellular neurofibrillar tangles of the hyper-phosphorylated protein tau (7). Endogenously formed Aβ peptides are largely composed of 39–43 residues, with the most common species containing either 40 (Aβ40) or 42 (Aβ42) residues. The two additional residues at the C terminus of Aβ42 compared with Aβ40 has emerged as a factor modulating the propensity of this peptide to aggregate and its associated toxicity, with Aβ42 being more aggregation prone than Aβ40. Furthermore, the ratio of Aβ42 to Aβ40 has been found to be higher in AD patients than in healthy controls (8, 9).

It is increasingly evident that AD is associated with neuronal damage resulting from the aggregation of the Aβ peptide, chiefly Aβ42, and also of tau, which is likely to occur as a downstream process. We use the term aggregate to refer to any species containing two or more Aβ42 peptides and oligomer to refer specifically to low relative molecular weight aggregate species containing typically 2–15 molecules (10). Recent evidence suggests that this toxicity is largely associated with low molecular weight oligomers rather than with monomeric species or with the mature amyloid fibrils (10–13). This observation indicates that a fruitful therapeutic strategy to reduce the toxicity associated with the aggregation process would be to target specifically the pathways that lead to the formation of such oligomeric toxic species rather than aim to arrest the fibril formation itself. A fundamental prerequisite to achieve such selective targeting is a detailed understanding of the molecular mechanism underlying fibril formation in the presence and absence of molecular species able to perturb the aggregation process. In this context, kinetic analysis in combination with experimental measurements is emerging as a powerful tool to provide a mechanistic description of the aggregation process and its inhibition (10, 14–19). Kinetic studies have shown *in vitro* that Aβ42 aggregation occurs by a double nucleation mechanism (10, 20), with the primary nucleation of monomers in solution being significantly slower than the secondary nucleation catalyzed by the surfaces of amyloid fibrils (10).

A wide range of molecules have been reported to influence the aggregation process of Aβ peptides, including small molecules, designed peptides, antibodies and other proteins (15, 19, 21–23).^3^ A crucial class of inhibitors in living systems is that of molecular chaperones, which in addition to their role in assisting protein folding and assembly (24, 25), are known to suppress aggregation induced by heat shock or other proteotoxic stresses (26–28), and play a key role in suppressing amyloid formation and promoting clearance of misfolded species (29, 30). Moreover, the chaperone αB-crystallin (HSPB5) is overexpressed in post mortem brains of AD patients and is co-localized with Aβ aggregates in eye lenses from such patients (31, 32), and in addition retards Aβ fibril formation *in vitro* (21, 22). Recent kinetic studies reveal that the capability of natural molecular chaperones to inhibit aggregation may involve the suppression of single specific steps in the aggregation process. For instance, a chaperone belonging to the Brichos family (19) has been found to suppress specifically the secondary nucleation step of Aβ42 aggregation.^3^ Despite the fact that such inhibition does not affect the total amount of mature fibrils that are eventually formed, the suppression of this specific step is highly efficient in decreasing the numbers of oligomers generated during the reaction and hence the toxicity associated with the aggregation process (33).^3^

DNAJB6 is a human molecular chaperone belonging to the Hsp40 heat shock protein family. This chaperone has been recently found to perturb the formation of fibrils by polyglutamine peptides (34), which are involved in neurodegenerative disorders such as Huntington disease (35, 36). In the present work, we show that DNAJB6 is a potent inhibitor of the aggregation of Aβ42, acting in a concentration-dependent manner at remarkably low stoichiometric ratios. We demonstrate by means of kinetic analysis and immunochemistry experiments that such high efficiency originates from the capability of DNAJB6 to sequester effectively the Aβ42 aggregates, which propagate the amyloid conversion reaction, thereby preventing their growth and limiting their ability to proliferate through secondary nucleation.

## EXPERIMENTAL PROCEDURES

### 

#### 

##### Peptides and Proteins

Aβ42. Human Aβ peptide, Aβ(1–42), UniProtKB ID P05067, residues 672–713, with an N-terminal methionine residue, also corresponding to residue 671 of APP, (MDAEFRHDSGYEVHHQKLVFFAEDVGSNKGAIIGLMVGGVVIA) was expressed recombinantly in *Escherichia coli* BL21 DE3 star PLysS and purified essentially as described previously (10, 18, 37) to obtain a pure monomers from which to initiate the aggregation reaction and achieve high reproducibility.

##### DNAJB6

Human DNAJB6b (isoform b, UniProt ID O75190–2) with a hexa-His tag was expressed recombinantly in *E. coli* ER2566 and purified as described previously (34) but with an additional washing step using 8 m urea during the affinity chromatography in order to remove bound bacterial proteins (38). Just prior to its use, DNAJB6 was dialyzed into the assay buffer (20 mm sodium phosphate buffer pH 8, 0.2 mm EDTA, 0.02% sodium azide) using Slide-A-Lyser MINI (Thermo Scientific, Rockford, IL).

##### αB-Crystallin

Human αB-crystallin (UniProtKB ID P02511) was recombinantly expressed and purified as previously described (39). The protein was desalted using PD10 desalting column, (GE Healthcare, Little Chalfont, UK) eluted in assay buffer, and concentrated, when necessary, by Nanosep 3 K Omega (Pall Life Sciences, Port Washington, NY), and stored at −20 °C until use.

##### Human Serum Albumin (HSA)

HSA (fatty acid free, 99% pure) was obtained from Sigma (Stockholm, Sweden) and purified as described previously (40).

##### Determination of Protein Concentration

Protein concentrations are reported as monomer equivalents for DNAJB6 and αB-crystallin, determined as the mean values between the values obtained from the Bradford assay (41, 42), using BSA as a reference, and the absorbance at 280 nm taken from their respective theoretical extinction coefficients. The concentration of Aβ42 was determined using absorbance at 280 nm of the collected monomer fraction, using ϵ = 1400 mol^−1^cm^−1^ as verified using amino acid analysis after acid hydrolysis.

##### Denaturing Electrophoresis

Denaturing gel electrophoresis was performed using precast 4–12% NuPAGE® SDS-PAGE Bis-Tris gels (Invitrogen, Stockholm, Sweden) according to the manufacturer's instructions. Samples were diluted with lithium dodecyl sulfate sample buffer (4×) (Invitrogen, Stockholm, Sweden) and electrophoresis was run in 50 mm MES buffer pH 7.3. Gels were silver-stained according to established protocols (43) for maximized sensitivity, and scanned on an Image Scanner III with the Labscan software (GE Healthcare LifeSciences, Uppsala, Sweden).

##### CD Spectroscopy

Ellipticity was recorded between 250 and 185 nm in a quartz (QS) cuvette with 10 mm path length at 37 °C using a Jasco J-815 CD spectrometer. The samples were stirred between measurements but not during measurement. The scanning rate was 50 nm/min, the digital integration time per data point (D.I.T.) was 8 s, the sensitivity was set to the standard value, and the reported data were averaged over three accumulations. The data were cropped at the wavelength where the voltage exceeded 800 V, and the background signal from the buffer (5 mm sodium phosphate buffer, pH 8.0 with 40 mm NaF) or the buffer including 0.05 μm DNAJB6 was subtracted. The Aβ42 concentration was 5 μm, and the DNAJB6 concentration 0.05 μm.

##### Aggregation Kinetics

Aggregation kinetics were monitored as described previously (18) using a Thioflavin T (ThT) fluorescence assay based on the enhanced quantum yield of ThT fluorescence as the dye binds to amyloid fibrils. All experiments were performed in the assay buffer (20 mm sodium phosphate buffer pH 8, 0.2 mm EDTA, 0.02% sodium azide) with 10 μm ThT in microplate wells (Mikroplate Corning 3881, 96-well, low-binding, half-area, Corning Incorporated Life Sciences, Acton, MA) with 70 μl solution per well

**FIGURE 1.**
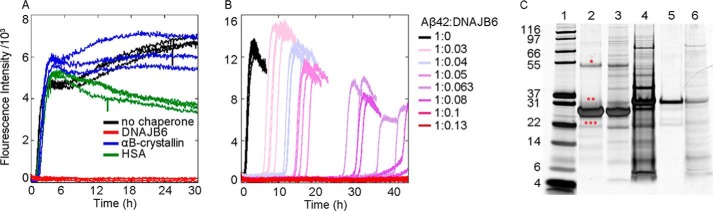
**Aβ42 aggregation in the absence and presence of the human molecular chaperone DNAJB6.** *A*, fibril formation by 3 μm Aβ42 solutions was monitored by following the increase in ThT fluorescence (*black*); the inhibitory effect of DNAJB6 (*red*) was compared with that of human αB-crystallin (*blue*) and HAS (*green*). Molar ratios between Aβ42 and the proteins were 1:0.1. *B*, aggregation reaction profiles of ThT fluorescence of Aβ42 in the absence (*black*) and in the presence of DNAJB6 at molar ratios of peptide to chaperone from 1:0.01 to 1:0.13, color coded as indicated to the *right*. 4 independent incubations per sample were performed. *C*, silver-stained SDS-PAGE shows the purity of the chaperone with and without the washing step with 8 m urea to remove proteins strongly bound to DNAJB6: *lane 1*, molecular weight marker (kDa); *lane 2*, urea-washed DNAJB6; *lane 3*, not urea-washed DNAJB6; *lanes 4* and *5*: first and second wash fraction (with and without urea, respectively) resulting in DNAJB6 in *lane 2*; *lane 6*, wash fraction without urea resulting in DNAJB6 in *lane 3*. The *asterisks* are positioned above bands indicating minor amounts of DNAJB6 degradation product (***), DNAJB6 monomers with His-tag (**), and DNAJB6 dimers (*).

Samples were prepared on ice in low binding tubes using freshly prepared monomeric Aβ42 at the final concentrations stated in the text and at least three measurements per sample were recorded. ThT fluorescence was recorded at 2-min intervals under quiescent conditions without agitation at 37 °C, using a Fluostar Omega or Optima plate reader (BMG Labtech, Offenburg, Germany) with a 440 nm excitation filter and a 480 nm emission filter. In Fig. 1, the total fluorescence intensity is shown, in the other figures the relative intensities normalized to 1 are presented.

##### Production of Aβ42 Seed Fibrils

To produce Aβ42 seed fibrils, 7 μm Aβ42 monomer with 10 μm ThT was incubated and fibril formation was monitored by ThT fluorescence for 2 h, at which point equilibrium was reached, and a suspension of 7 μm seed fibrils was obtained. The seed fibrils were sonicated for 2 min.

##### Kinetic Analysis of Unseeded Reactions

The time evolution of the total fibril mass *M(t*) in the presence of both primary and secondary nucleation events is described by the integrated rate law in Equation 1 (10, 44, 45):

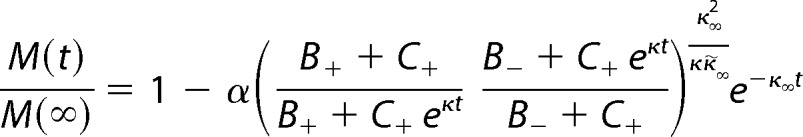
 where the kinetic parameters *B*_±_, *C*_±_, κ, κ_∞_, and κ̃_∞_ are functions of combinations of the microscopic rate constants k_+_k_2_ and k_n_k_2_, where k_n_, k_+_, and k_2_ are the primary nucleation, fibril elongation, and secondary nucleation rate constants, respectively.

As DNAJB6 could alter the global aggregation profile by inhibiting one or more of the individual microscopic events, we have carefully identified the microscopic steps that are inhibited by the molecular chaperone. This objective was achieved by applying Eq. 1 to describe the global profiles of the unseeded reactions shown in Fig. 3, *A* and *B*, and comparing the set of microscopic rate constants k_+_k_2_ and k_n_k_2_ required to describe the time evolution of the fibril formation in the absence and presence of DNAJB6.

##### Filter Trap Assay

3 μm Aβ42 solutions were prepared as for the kinetic aggregation studies, in the absence and presence of DNAJB6 at the final concentrations as stated, and the time evolution of the ThT fluorescence was recorded. At selected time positions, as indicated by the asterisks, and at the end of the experiment, aliquots of sample were extracted, mixed with PBS containing 1% SDS, and added to a cellulose acetate membrane with 0.2 μm pores (GE Healthcare, Uppsala, Sweden) with a vacuum device. The membranes were washed twice with PBS containing 1% SDS, and then with PBS without SDS. The membrane was blocked with nonfat dry milk in PBS with 0.1% Tween-20 for 1 h, and thereafter primary polyclonal rabbit antibodies for DNAJB6 (Innovagen, Lund, Sweden) diluted 1:1000 and Aβ, 6E10 monoclonal mouse (Covance, Princeton, NJ) diluted 1:1000, were added to the membrane followed by an incubation for 1 h. After washing in PBS with and without Tween-20, the membranes were incubated for 1 h with secondary antibodies from LiCORE: IRDye 800CW goat anti-rabbit and IRDye 680RD goat anti-mouse in nonfat dry milk in PBS with Tween-20. The membranes were washed, dried, and stored in the dark until analyzed. The fluorescence emission of the membranes was imaged at two wavelengths using an Odyssey CLX IR imaging system (LICOR, Cambridge, UK).

## RESULTS

### 

#### 

##### DNAJB6 Inhibits Fibril Formation by the Aβ42 Peptide

We investigated the effect of DNAJB6 on fibril formation by highly purified recombinant human Aβ42 peptide under quiescent conditions by monitoring the intensity of ThT fluorescence reporting on the formation of amyloid fibrils. At the concentration of Aβ42 used as a reference point in this work (3 μm), we observed a reaction half time, defined as the time for half of the initial monomeric pool to form ThT-sensitive aggregates, t½ ≈1.5 h (Figs. 1, *A* and *B* and 3), in close agreement with an earlier report (10). However, in the presence of even a sub-stoichiometric concentration of the molecular chaperone DNAJB6 at a molar ratio of 1:0.1 Aβ42 to DNAJB6, the aggregation process was fully suppressed for 30 h (Fig. 1*A*) with no observable ThT signal being detected over this length of time. The concentrations and molar ratios are given throughout this work as monomer equivalents, since DNAJB6 is an oligomeric protein with varying numbers of monomers per oligomer; a representative SDS-PAGE gel of the highly purified protein is shown in lane 2 in Fig. 1*C*. Addition of a non-chaperone protein, human serum albumin (HSA), at a molar ratio of 1:0.1 did not affect the rate of fibril formation by Aβ42. Moreover, addition at the same molar ratio (1:0.1) of αB-crystallin, a member of the family of small heat shock proteins previously shown to inhibit Aβ42 peptide aggregation (21) at higher concentrations, did not affect the fibril formation process when used under conditions where the Aβ42 was in significant excess. Hence, DNAJB6 exhibits a particularly potent inhibitory effect with respect to other proteins.

To analyze the effect of DNAJB6 on Aβ42 fibril formation in more detail and determine its mechanism of action, we performed a series of kinetic experiments by varying systematically the DNAJB6 concentration while maintaining the concentration of Aβ42 fixed (Fig. 1*B*). These results show that DNAJB6 causes a concentration-dependent retardation of Aβ42 aggregation, and is effective already at highly sub-stoichiometric concentrations (1:0.03 molar ratio). The t_1/2_ increases progressively from ca. 1.7 h in the absence of chaperone to 36 h at 1:0.08 Aβ42 to DNAJB6 molar ratio. At DNAJB6 concentrations above 0.3 μm, corresponding to a molar ratio of Aβ42 to DNAJB6 of 1:0.1, fibril formation was not observed during the time-frame of the experiment (45 h).

**FIGURE 2.**
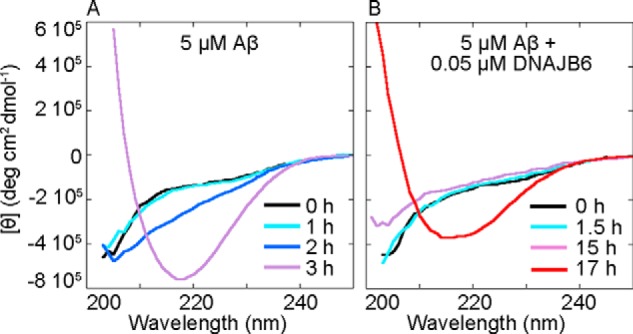
**DNAJB6 delays the conversion of Aβ42 from unstructured monomers to β-sheet structures.** Far-UV CD spectra of 5 μm Aβ42 solutions showing the appearance of the characteristic β-sheet structure signal with a minimum intensity at c.a. 218 nm at different time points at 37 °C in *A* the absence and *B*, the presence of 0.05 μm DNAJB6. In *A* the signal from the buffer has been subtracted, in *B* the signal from the buffer including 0.05 μm DNAJB6 has been subtracted.

The analysis of the rates of fibril formation using ThT fluorescence is supported by measurements using far-UV CD spectroscopy, which can detect the characteristic β-sheet structure of amyloid fibrils generated by the self-assembly of the peptide even in the absence of ThT reactive species. The spectra of Aβ42 change from those characteristic of the disordered structure of the monomer to the β-sheet structure of the fibrils, which exhibit a characteristic minimum at around 218 nm. At a 1:0.01 Aβ42:DNAJB6 molar ratio, DNAJB6 delays the β-sheet structure formation of a 5 μm Aβ42 solution such that the time required to reach the end point of the aggregation reaction increases from 3 h (Fig. 2*A*) to 17 h (Fig. 2*B*), in excellent agreement with the ThT data shown in Fig. 1*A*, thereby confirming that the formation of fibrils is delayed in the presence of DNAJB6.

##### Kinetic Analysis of Unseeded and Seeded Reactions Reveals that DNAJB6 Suppresses Both Primary and Secondary Nucleation Events

**FIGURE 3.**
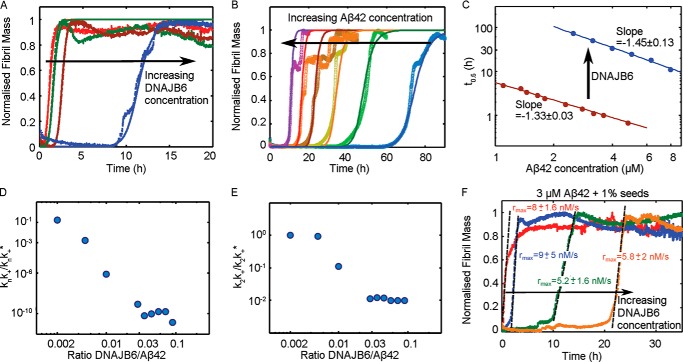
**Kinetics of Aβ42 aggregation in the presence of DNAJB6.** Reaction profiles for ThT fluorescence for unseeded aggregation of (*A*) 3 μm Aβ42 solutions in the absence of DNAJB6 (*red*) and with DNAJB6 present at a molar ratio of peptide to chaperone of 1:0.001 (*green*), 1:0.005 (*maroon*) and 1:0.01 (*purple*), and (*B*) Aβ42 solutions in the concentration range 2.2–8.0 μm in the presence of 0.225 μm DNAJB6. The lines in *A* and *B* represent the integrated rate law for Aβ42 aggregation fitted to the experimental data. *C*, half-times as a function of the initial Aβ42 concentration in the presence and absence of DNAJB6. Note the change of exponent from −1.33 to −1.45 induced by the presence of DNAJB6, showing the capability of the chaperone to suppress primary nucleation events. *D* and *E*, set of microscopic rate constants corresponding to the kinetic analysis shown in *A* and *B* normalized to the values in the absence of chaperone as a function of DNAJB6:Aβ42 molar ratios. For unseeded reactions the inhibition of primary nucleation events (k_n_k_+_/k_n_k*_+_ (*D*)) is significantly larger with respect to secondary nucleation events (k_2_k_+_/k_2_k*_+_ (*E*)). *F*, reaction profiles of 3 μm Aβ42 solutions with 1% seeds in the absence of DNAJB6 (*red*) and with 1:0.01 (*blue*), 1:0.02 (*green*), and 1:0.05 (*orange*) monomer equivalents of DNAJB6 showing the capability of the chaperone to suppress secondary nucleation events.

To gain insight into the microscopic inhibition mechanism of the chaperone, we performed kinetic studies of Aβ42 aggregation reactions at different DNAJB6 and Aβ42 concentrations (15) (Fig. 3, *A* and *B*). Analysis of such kinetic data has shown that the scaling of the half-times of the aggregation reaction profiles as a function of the total protein concentration follows a power law whose exponent contains important information about the microscopic events underlying the macroscopic aggregation behavior (16, 44, 45). From the measured half-times of the Aβ42 aggregation data obtained for different peptide concentrations, we obtain a scaling exponent of −1.33 in the absence of DNAJB6 (Fig. 3*C*), consistent with our previous findings (10). This exponent originates from the situation (10) where secondary nucleation events dominate the generation of new fibrils, although primary nucleation events are still present and reduce the exponent from the limiting value of −3/2, which corresponds to pure secondary nucleation events (10, 16, 44, 45). In the presence of DNAJB6 (Fig. 3*A*), the lag times increase and the scaling exponent decreases to −1.45 (Fig. 3*C*), an observation that suggests that, under these conditions, the action of the molecular chaperone originates mainly through its inhibitory effect on primary nucleation reactions, thereby increasing the relative importance of secondary nucleation events.

This conclusion is confirmed by the detailed analysis of the effects of DNAJB6 on the microscopic events involved in the aggregation of the Aβ42 peptide. To this effect, to obtain the rate constants describing the microscopic events of primary and secondary nucleation and fibril elongation, we performed global fits to the integrated rate laws for filamentous growth (10, 20). In this approach, a set of kinetic profiles recorded at different initial concentrations of soluble peptide are fitted to a single rate law with a single set of rate constants, thereby placing strong constraints on the fitting parameters. We then compared the microscopic rate constants required to describe the time evolution of fibril formation in the presence of DNAJB6 with the set of rate constants estimated previously in the absence of the chaperone (10). This analysis shows that the molecular chaperone inhibits both primary and secondary nucleation events (Fig. 3, *D* and *E*), but that the inhibitory effect on primary nucleation events is significantly greater. Indeed, the combination of kinetic constants describing the primary pathway (17, 44, 46), k_n_k_+_ decreases by a remarkable 10 orders of magnitude (Fig. 3*D*), while the corresponding set of rate constants governing the secondary pathway, k_2_k_+,_ decreases only by 2 orders of magnitude (Fig. 3*E*) as the molecular chaperone is introduced into the reaction mixture. This result is therefore fully consistent with the change of the scaling exponent toward the theoretical value corresponding to the presence of secondary nucleation events only (Fig. 3*C*).

Consideration of the stoichiometry of the system suggests that we can exclude the possibility that the inhibitory effect discussed above is due simply to binding between DNAJB6 and monomeric Aβ42. Indeed, even if several Aβ42 peptides (M_w_ = 4.5 kDa) could bind to each DNAJB6 monomer (M_w_ = 27 kDa) at a 1:0.01 molar ratio, this effect would reduce the free Aβ42 monomer concentration only by a few percent, which would have only a minor impact on the observed aggregation rate. We can conclude, therefore, that DNAJB6 affects primary nucleation processes by interacting with the aggregated species that result from the nucleation process, hence suppressing their growth into longer fibrils that are required for the effective catalysis of the aggregation reaction by secondary processes (10).

**FIGURE 4.**
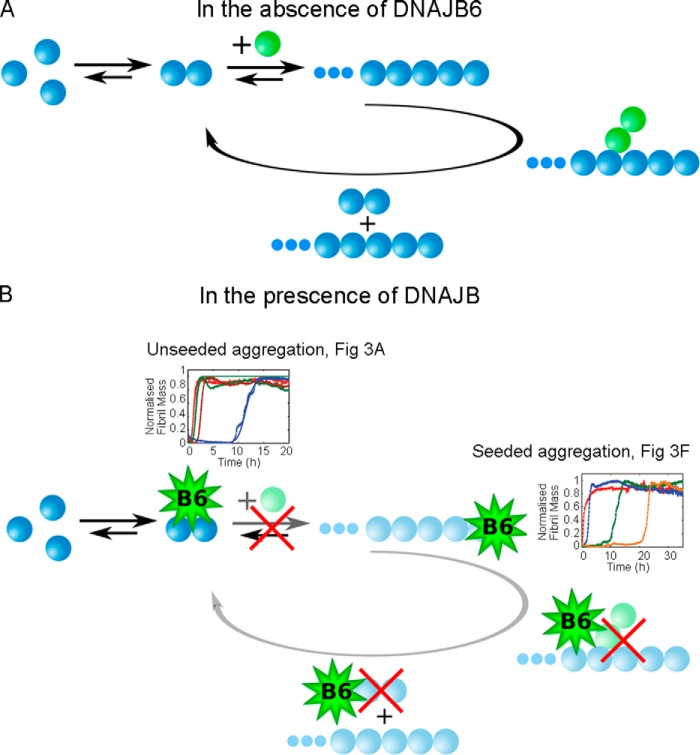
**Prevention of nucleation pathways by the chaperone DNAJB6.** Schematic diagram showing (*A*) the molecular pathways involved in Aβ42 aggregation and (*B*) the proposed mechanism by which DNAJB6 inhibits the aggregation reaction. As indicated in faded colors in *B*, the interactions between the chaperone and the Aβ42 growing aggregates inhibit both primary (as seen in Fig. 3*A*) and secondary (as seen in Fig. 3*F*) nucleation pathways.

To investigate further the inhibition of the elongation and secondary processes by DNAJB6, we performed seeded aggregation experiments. Under such conditions, where the reaction is initiated by adding a well-defined concentration of pre-formed seed fibrils to solutions of the monomeric peptide, the overall kinetics can become largely independent of the nucleation processes since the presence of pre-formed fibrils circumvents the nucleation barrier (47). As such, seeded experiments can be a powerful tool to probe the changes in a specific microscopic process in the aggregation pathway, the elongation step. The results, shown in Fig. 3*F*, indicate that DNAJB6 is also effective in inhibiting fibril formation in a concentration-dependent manner when the process is initiated by the addition of 1% *w*/*w* of pre-formed fibrils to the reaction mixture. Moreover, the presence of the molecular chaperone restores the lag-phase which is very short in the absence of DNAJB6. This observation indicates that DNAJB6 interacts not only with aggregates formed as a result of primary nucleation but also with the structures originating from secondary nucleation, whose formation is catalyzed by the surface of seed fibrils, and prevents the elongation and the autocatalytic multiplication of aggregated Aβ42. Furthermore, it is also likely that DNAJB6 interacts with the added seeds themselves since their elongation is suppressed. Taken together, the data indicate that DNAJB6 reduces the rate of fibril formation by inhibiting all the main classes of events in the reaction process, primary and secondary nucleation and elongation; moreover, the kinetic analysis shows that specific interactions with species formed after primary and secondary nucleation are the major origin of the very large magnitude of this inhibition effect (Fig. 4).

We note that the molecular chaperone delays but does not suppress completely the fibril formation process. In particular, under seeding conditions, the reaction profiles exhibit significantly different lag-phases in the presence and absence of DNAJB6 but very similar growth phases (Fig. 3*F*). This observation suggests that during the initial phases of aggregation, the molecular chaperone interacts with all aggregated species, but that after a given incubation time, which is proportional to the initial chaperone concentration (Fig. 3*F*), the inhibitory effect disappears. This observation indicates that at this incubation time the chaperone is no longer effective, suggesting the possibility that it could be depleted from the solution, while Aβ42 is still present as free monomers able to participate in the aggregation reaction.

##### DNAJB6 Sequesters Growing Aβ42 Species and Is Incorporated into the Fibrils

**FIGURE 5.**
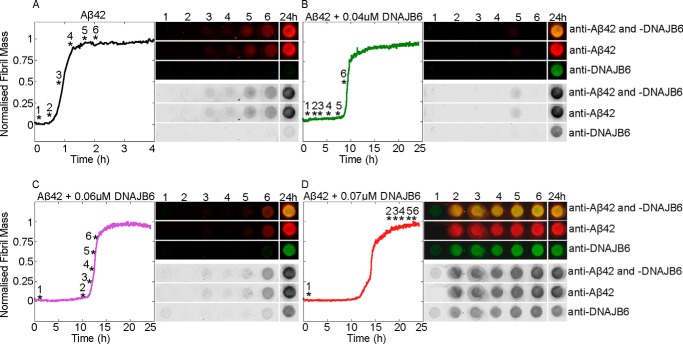
**Incorporation of DNAJB6 into fibrils.** Fibril formation by 3 μm Aβ42 solutions in the absence (*A*) and presence of 0.04 μm (*B*), 0.06 μm (*C*), and 0.07 μm (*D*) DNAJB6 were incubated at 37 °C and monitored by ThT fluorescence. Six samples (marked with asterisks) taken at six time points during the fibril formation process, and one sample withdrawn at the end of the experiments after 24 h, were examined. SDS-insoluble species were trapped on a cellulose acetate membrane, incubated with antibodies against Aβ42 and DNAJB6, and detected simultaneously with secondary antibodies labeled with different chromophores.

**FIGURE 6.**
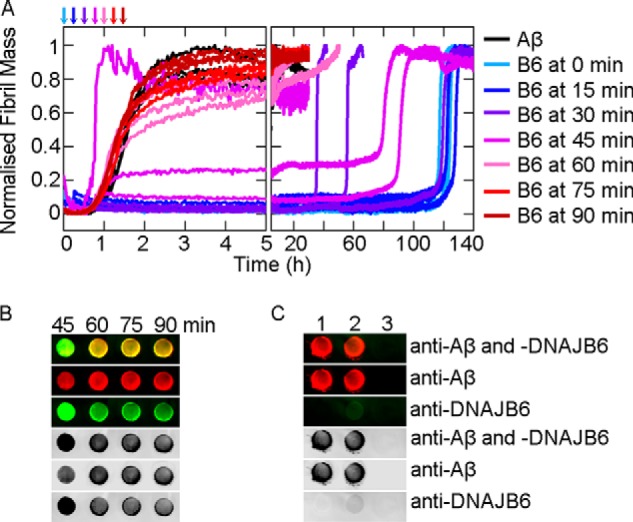
**Arrest of ongoing reactions by binding of DNAJB6 to Aβ42 aggregates.** *A*, 0.3 μm DNAJB6 was added to 3 μm Aβ42 solutions at 0, 15, 30, 45, 60, 75, or 90 min after the initialization of fibril formation at 37 °C. *B*, SDS-insoluble fibrils from the reactions in *A* corresponding to incubation times of 45, 60, 75, and 90 min were withdrawn after 35 h in the platereader and trapped on a cellulose acetate membrane, incubated with antibodies against Aβ42 and DNAJB6, and detected simultaneously with secondary antibodies labeled with different chromophores. *C*, control experiments for the dual immunodetection: 1: 3 μm Aβ42 fibrils alone, 2: 3 μm Aβ42 fibrils mixed with 0.3 μm DNAJB6 just prior to trapping, and 3: 0.3 μm DNAJB6 alone.

To test whether or not aggregating Aβ42 is able to deplete DNAJB6 from the solution over time, we performed immunoblot analysis of samples taken at different time points during the Aβ42 aggregation reactions performed in the absence (Fig. 5*A*) and presence of DNAJB6 at different concentrations (Fig. 5, *B–D*). The resulting fibrils, which are SDS-insoluble, were trapped using a cellulose acetate membrane and the composition of the trapped material was probed using antibodies against Aβ42 and DNAJB6. Remarkably, under conditions where the Aβ42 fibrils were formed in the presence of DNAJB6, the chaperone was also detected at the filtration membrane, indicating that it is strongly associated with the SDS-insoluble fibrils. We then evaluated the capability of DNAJB6 to block on-going aggregation reactions by adding aliquots of the chaperone at different time points during the incubation (Fig. 6*A*). Inhibition of the aggregation kinetics was observed under the conditions used here when DNAJB6 was added during the first 30 min of the lag-phase; but after 45 min, when the ThT signal starts to increase above the background noise level (when just over 5% of the Aβ42 monomers had converted to fibrils), addition of DNAJB6 showed no inhibition of fibril formation.

DNAJB6 is therefore not able to suppress the aggregation reaction after a critical concentration of aggregates, sufficient to deplete the chaperone from solution, has been formed. These results confirm the existence of a strong interaction between DNAB6 and Aβ42 aggregates and corroborate the conclusion that the microscopic mechanism of inhibition of Aβ42 aggregation under these conditions is due to sequestration by the chaperone of the growing aggregate species. We explicitly verified the capability of DNAJB6 to bind to Aβ42 aggregates during the course of the aggregation reaction through immunochemical analysis of the SDS-insoluble species obtained at the end of the reactions where DNAJB6 was added after 45, 60, 75, and 90 min (Fig. 6*B*). The results show that the maximum amount of DNAJB6 trapped in the fibrils was observed when the DNAJB6 was added after 45 min. When the DNAJB6 was added at later time points, corresponding to ∼15, 30, and 50%, respectively, of monomer conversion into fibrils, a progressive decline in the bound DNAJB6 was detected with the SDS-insoluble species. In addition, in the absence of fibrils no DNAJB6 was trapped on the filtration membrane, whereas pre-formed Aβ42 fibrils were found to bind DNAJB6 only to a small extent (Fig. 6*C*). The latter result indicates that the incorporation of the chaperone occurs during fibril growth.

**FIGURE 7.**
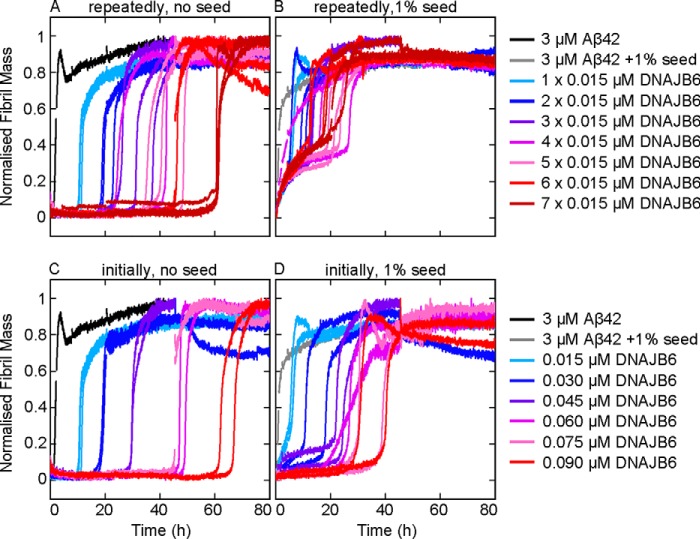
**Extension of the inhibitory effect for longer times by multiple additions of DNAJB6.** Aliquots of DNAJB6, each giving a concentration of 0.015 μm, were added repeatedly into the (*A*) unseeded and (*B*) seeded aggregation reactions, from one to seven times at the time points 0, 0.5, 1, 2, 4, 8, and 20 h. For comparison, the same total amount of DNAJB6 was added at the beginning of the experiment in the (*C*) unseeded and (*D*) seeded aggregation reactions.

The depletion of soluble DNAJB6, due to the high affinity and the low dissociation rate of the binding to the accumulating oligomers and fibrils, thus explains the observed loss of the inhibitory effect at the end stages of the aggregation reaction. Moreover, this model predicts that a continuous introduction of small amounts of chaperone during the aggregation reaction should prolong the inhibitory effect. This hypothesis was confirmed by performing aggregation experiments where low concentrations of the chaperone were repetitively added at different time-points during the aggregation reaction (Fig. 7). The results show that the addition of DNAJB6 during the aggregation process prolongs the inhibitory effect both in unseeded (Fig. 7*A*) and in seeded reactions (Fig. 7*B*). For seeded reactions, however, the inhibitory effect is substantially larger if DNAJB6 is added as a single aliquot at the beginning of the reaction (Fig. 7*D*) relative to the addition of fractions of the same amount at different time-points (Fig. 7*B*). This behavior can be rationalized by noting that a larger amount of molecular chaperone is likely to be required at the beginning of the reaction to sequester and deactivate the quantity of seed fibrils introduced into the reaction mixture. By contrast, for unseeded reactions, the inhibitory effect was found to be closely similar when the same total amount of DNAJB6 was added as a single aliquot at the beginning of the reaction (Fig. 7*C*) or added at different time points during the reaction (Fig. 7*A*). Also, the fact that a specific amount of DNAJB6 is required for sequestration of a specific quantity of aggregated Aβ42 rather than a specific threshold concentration of DNAJB6, indicates that the concentration of the chaperone, despite being very low relative to that of the client peptide, is larger than the equilibrium dissociation constant of binding between the chaperone and the Aβ42 species. As a consequence, DNAJB6 is able to sequester an amount of oligomers that is proportional to the amount of DNAJB6. This observation explains the finding that there is a point in time (between 30 and 45 min in Fig. 6*A*) where addition of DNAJB6 no longer inhibits the fibril formation: the quantity of aggregated and growing Aβ42 species exceeds that of the chaperone.

**FIGURE 8.**
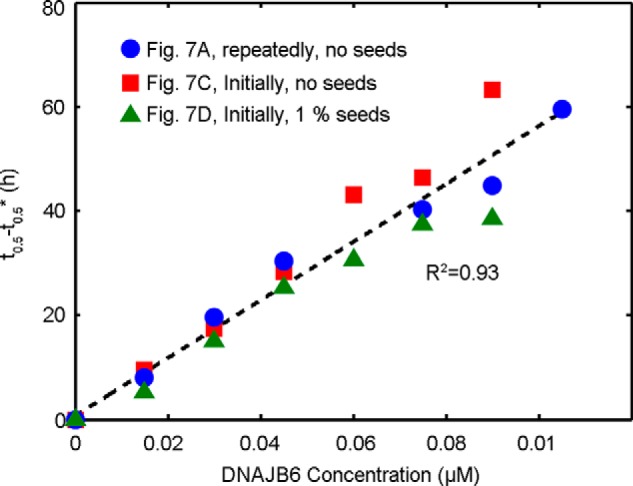
**Extension of lag-phase by sequestering of growing Aβ42 aggregates by DNAJB6.** The increase in the half-times of the reaction profiles shown in Fig. 7 as a function of DNAJB6 concentration. The symbol t_0.5_* represents the half-time of the aggregation reaction in the absence of chaperone. The linear increase observed for both unseeded and seeded reactions suggests that DNAJB6 binds to the growing aggregates with high affinity as described in the main text.

To generate a concentration, ΔP, of aggregates sufficient to replace those sequestered by the chaperone, an additional time Δt = ΔP/r_0_ is required, where r_0_ is the nucleation rate at the early stage, which is to a good approximation a constant since the monomer depletion at this time point is negligible (44). Such a mechanism should therefore result in an increase in both the lag-time and the half-time of fibril formation, which is linearly proportional to the concentration of the chaperone. This behavior is observed for all the sets of data of both the unseeded and seeded reactions (Fig. 8).

Taken together, all the data indicate clearly that the molecular chaperone achieves its remarkably effective and sub-stoichiometric inhibition by sequestering the Aβ42 oligomeric species (Fig. 4) generated through both primary and secondary nucleation.

## DISCUSSION

DNAJB6 is a member of the Hsp40 family of molecular chaperones involved in a wide range of cellular events. DNAJB6 has been identified as a particularly powerful suppressor of polyglutamine aggregation and of its associated toxicity in a screen that compared different types of chaperones, namely HSPA (Hsp70), HSPH (Hsp110), and DNAJ (Hsp40) (48). DNAJB6 is effective at sub-stoichiometric levels both *in vitro* and in human cell lines, suggesting that DNAJB6 is a peptide chaperone that interacts with polyglutamine peptides released from the proteasome (34, 49).

In the present work, we have demonstrated that DNAJB6 inhibits the aggregation of another and even more aggregation-prone peptide, Aβ42, at remarkably low sub-stoichiometric molar ratios of chaperone to peptide and identified the molecular inhibition mechanism underlying this potent activity, which is notably significantly larger than the inhibitory activity of human αB-crystallin and HSA at corresponding molar ratios (Fig. 1). Analysis of the aggregation kinetics shows that DNAJB6 can impede the growth and the autocatalytic self-multiplication of Aβ42 fibrils in both unseeded and seeded reactions, thereby showing that inhibition of the reaction must involve suppressing both the nucleation and the growth processes involved with Aβ42 aggregation reaction (Fig. 4). Stoichiometric considerations suggest that the chaperone interacts with aggregated forms of the peptide rather than with monomers, a mechanism confirmed by immunoblot analysis, which indicates that DNAJB6 is incorporated into the growing fibrils (Figs. 5 and 6). As a consequence, at low sub-stoichiometric values the chaperone is depleted from the solution, leading to the disappearance of its inhibitory effect over time. This conclusion was confirmed by kinetic experiments in which the introduction of DNAJB6 into the system at regular time intervals prolongs the inhibitory effect (Fig. 7). In particular, an equivalent inhibitory effect was observed when the same total amount of DNAJB6 was added as a single aliquot at the beginning of the reaction (Fig. 7*C*) as when it was added in a progressive manner at different time points (Fig. 7*A*) over the course of the reaction. This observation indicates further that DNAJB6 binds with high affinity to the growing aggregates, a finding supported by the linear increase in the duration of the lag-phase as a function of chaperone concentration in reaction profiles at both unseeded and seeded conditions (Fig. 8).

The specific nature of the interactions between the chaperone and specific types of protein aggregates at the molecular level, as well as the correlation with the chaperone structure, remain to be elucidated. DNAJB6 has a conserved N-terminal HSPA-interacting J-domain, characteristic of all DNAJ protein family members, a G/F-rich central domain and a C-terminal segment which is inferred to be a peptide-binding domain. An S/T-rich region in the beginning of the C-terminal domain, conserved only among DNAJB6 and DNAJB8 proteins, was observed to be important for the suppression of the aggregation of Huntingtin exon-1 and for the formation of oligomers (48). In contrast with DNAJB6, the canonical Hsp40 chaperone DNAJB1 has by itself an insignificant effect on the aggregation kinetics of Aβ42, although it acts to enhance the aggregation inhibition effect of an Hsp70 homolog (50). DNAJB1 also has only a very minor effect on the aggregation of polyglutamine peptides (34, 48), emphasizing the large difference in specificity and functionality among the various members of the large and highly diverse DNAJ-protein family (51). Since the largest difference in sequence between DNAJB1 and DNAJB6 is in the C-terminal domain, this observation supports a possible dominant role of this domain in the interactions with misfolded species, which are likely to be mediated by hydrophobic forces. Indeed, charged hydrophilic proteins with both negative and positive net charges, including proteins with a similar net charge to that of DNAJB6, are much less potent inhibitors of amyloid formation than DNAJB6 (52). This implies that long-range electrostatic effects play only a minor role in the specific interactions with initially formed aggregates. Interactions with solvent exposed hydrophobic residues in the aggregated Aβ42 species (53, 54), are therefore likely to underlie the sequestration of the initial products of the primary and secondary nucleation reactions of Aβ42 by DNAJB6, a process which may involve regions of DNAJB6 that can be buried in oligomeric states of DNAJB6 itself. The prevalence of hydroxyl groups in the conserved S/T region is another intriguing feature that may provide high capacity hydrogen bonding to edge strands of growing aggregates.

We conclude that DNAJB6 inhibits Aβ42 fibril formation at low sub-stoichiometric ratios by interacting selectively with the most reactive species present in the system, in particular by sequestering the aggregated species generated by primary or secondary nucleation. This result highlights an intriguing feature of the activity of a natural molecular chaperone against misfolding events and associated reactions, suggesting that the roles of chaperones are not limited to the sequestration of single monomeric unfolded conformations, but can involve multiple interactions with aggregated species.

## Footnotes

3 Cohen, S. I. A., Arosio, P., Presto, J., Kurudenkandy, F. R., Biverstal, H. *et al* 2014, submitted publication.

## Footnotes

2 The abbreviations used are:

AD: Alzheimer disease
Aβ42: amyloid-beta peptide
ThT: thioflavin T
HSA: human serum albumin.
